# Editorial: International evidence on predictors and outcomes of awareness of age-related change

**DOI:** 10.3389/fpsyt.2023.1128531

**Published:** 2023-01-24

**Authors:** Serena Sabatini, Hans-Werner Wahl, Roman Kaspar, Jonathan Huntley

**Affiliations:** ^1^School of Medicine, University of Nottingham, Nottingham, United Kingdom; ^2^Psychological Aging Research, Heidelberg University, Heidelberg, Germany; ^3^Cologne Center for Ethics, Rights, Economics, and Social Sciences of Health, University of Cologne, Cologne, Germany; ^4^Experimental Psychology, University College London, London, United Kingdom

**Keywords:** subjective aging, gains and losses, developmental outcomes, health, personality, cross-cultural, global aging

## Introduction

The concept of Awareness of Age-Related Change (AARC) was introduced in 2010 to capture the extent to which people become aware of and attribute the changes they experience in key life domains such as health and physical functioning, cognitive functioning, interpersonal relationships, socio-cognitive and socio-emotional functioning, and lifestyle and engagement to aging (1, 2). Importantly, and in contrast to the mostly unidimensional measures in the area of subjective aging, AARC builds on a multidimensional approach by considering AARC-gains and AARC-losses, thus following an essential tenet of lifespan developmental psychology. Assessing levels of awareness of positive (AARC-gains) and negative (AARC-losses) age-related changes is useful to better understand individuals' behaviors and risk of poor current and future health. Indeed, individuals reporting fewer AARC-gains and more AARC-losses engage less frequently in healthy and adaptive behaviors and score more poorly on measures of mental, physical, and cognitive health (3–6). Although evidence on AARC is rapidly increasing, studies have mainly been conducted in the US, Germany, and the UK. The goal of this Research Topic has been to add new empirical insights on AARC by presenting studies undertaken in Australia, Norway, Burkina Faso, China, Germany, and the U.S. to enrich the data portfolio related to AARC and broaden insights on its predictors and outcomes. Some of the studies included in the Research Topic advanced evidence on both AARC predictors and outcomes; they will therefore be discussed in both the following sections.

## Predictors of awareness of age-related change

Previous evidence suggests that levels of AARC-gains and AARC-losses may be the result of the interaction between the objective changes individuals experience (e.g., physical illness) and how they evaluate and address them based on personal characteristics (e.g., tendency to ruminate over losses) and age-related beliefs (e.g., interpreting all losses as being inevitable in older age). Existing evidence is further supported and enriched by the findings of this Research Topic. Testad et al. found, in a sample of 1,510 Norwegian participants aged 50+, that men and older people experience lower AARC-gains. Participants who obtained a university education, were employed, married/ in a civil partnership/ co-habiting reported lower AARC-losses.

Similarly, Schönstein et al. found, among 3,028 people aged 40+ and living in Burkina Faso, a quite different political-cultural context than that of Testad et al., that older age and higher education were associated with fewer AARC-gains and more AARC-losses. However, being male was associated with higher AARC-gains. The contrast in the results of these two studies may be explained by cultural differences between Norway and Burkina Faso in the health status and subjective perceptions of aging of men and women.

Wettstein et al. used a longitudinal approach to explore the extent to which chronological age, personality, and age stereotypes influence AARC-gains and AARC-losses over 5 years in 423 German participants aged 40–98. Over time, AARC-gains declined whereas AARC-losses remained fairly stable, further suggesting that fewer AARC-gains are experienced as one's age increases. Nonetheless, results suggest that higher AARC-gains can be better maintained when individuals have low neuroticism, high openness, high conscientiousness, and positive age stereotypes. High neuroticism and negative age-related stereotypes, in contrast appeared to draw attention to AARC-losses.

Dunsmore and Neupert also investigated the link between personality and AARC in a sample of 296 Americans aged, on average, 64.7 years (SD = 4.36 years; range: 60–90 years). More specifically, they explored the attenuating/exacerbating role personality traits may have on the cross-sectional link between having arthritis and lower AARC-gains and higher AARC-losses. They found that when people with arthritis score higher on agreableness, higher on conscientiousness, or lower on neuroticism, their levels of AARC-gains were higher. This finding suggests that personal characteristics can buffer the negative impact age-related illnesses have on perceptions of aging.

Finally, Rupprecht et al. explored, in a sample of 1,612 German individuals aged 16–93 years, whether life events over the past 2 years are related to AARC-gains and AARC-losses. Specific family-related life events, such as entering a new romantic relationship, were related to higher AARC-gains whereas specific health-related events, such as hospital stays, were related to higher AARC-losses.

## Outcomes of awareness of age-related change

While poor mental and physical health have been widely documented as outcomes of more negative AARC, less is known about cognitive functioning. Schönstein et al. found, in a sample of 3,028 individuals aged 40 and over and living in Burkina Faso, that lower AARC-gains and higher AARC-losses were associated at the cross-sectional level, in addition to fewer symptoms of depression and poorer cognitive functioning. Results are consistent with previous evidence from US and German samples (7, 8).

In some contrast, Testad et al. found that, in a sample of Norwegian individuals aged 50+, both higher AARC-gains and higher AARC-losses were related cross-sectionally to poorer performance on cognitive reasoning and this link is in line with what was found among UK individuals aged 50+ (5). Longitudinal studies may be needed to elucidate the associations of AARC-gains and AARC-losses with additional facets of cognitive functioning.

Wilton-Harding et al. found, in a sample of 152 Australian adults aged 53–86 years, that higher AARC-gains and lower AARC-losses were associated with lower negative affect and higher vitality (e.g., feeling alert and awake). Using a micro-longitudinal study design, the authors also found that higher concurrent AARC-gains buffered the adverse impact of high AARC-losses on negative affect.

Moreover, Schlomann et al. explored for the first time whether AARC is related to use of smartphone and mobile internet and attitudes toward technology. The study used data collected in 2020 in a sample of 557 German residents aged 42–94 years. Higher AARC-gains and lower AARC-losses were related to greater use of technology and more positive attitudes toward use of technology.

Finally, Zhang and Wood were the first to explore the psychometric properties of the AARC questionnaire in a sample of 421 teachers (Mean age = 42.4 years; SD = 8.5 years; range: 20–65 years) in China, as well as to explore the associations of levels of AARC-gains and AARC-losses with proactivity. They found that lower levels of AARC-gains and higher levels of AARC-losses are associated with reduced proactivity.

Taken together findings from studies included in this Research Topic suggest that, across a range of countries, people with certain socio-demographic characteristics (e.g., less education); health conditions (e.g., age-related illnesses such as arthritis), personality traits (e.g., neuroticism), and less positive age stereotypes report fewer AARC-gains and more AARC-losses. Due to lower AARC-gains and higher AARC-losses these people are at higher risk of poorer health and functioning. Importantly, antecedents were different for AARC-gains and AARC-losses, as were consequences for health, and AARC-gains and AARC-losses interacted in defining late-life wellbeing. As AARC-gains and AARC-losses are somewhat influenced by some potentially modifiable factors such as endorsement of negative age-stereotypes, as this Research Topic has shown, programs promoting more positive and realistic expectations regarding aging may result in higher AARC-gains and lower AARC-losses and consequently better health in older age (9).

## Author contributions

SS took the lead in writing the Editorial. H-WW, RK, and JH co-edited the paper. All authors contributed to the article and approved the submitted version.
